# Implementation of longitudinal insulin kinetic studies in busy field settings in Uganda: experience from the “glucose metabolism changes in Ugandan HIV patients on dolutegravir based anti-retroviral therapy” (GLUMED study)

**DOI:** 10.11604/pamj.2023.44.184.39734

**Published:** 2023-04-19

**Authors:** Frank Mulindwa, Jean-Marc Schwarz, Nele Brusselaers, Simon Dujanga, George Yendewa, Barbara Castelnuovo

**Affiliations:** 1 Makerere University Infectious Diseases Institute, Capacity Building Unit, Kampala, Uganda,; 2 Global Health Institute, Antwerp University, Antwerp, Belgium,; 3 University of California San Francisco, School of Medicine, California, United States of America,; 4 Department of Basic Sciences, Touro University California College of Osteopathic Medicine, Vallejo, California, United States of America,; 5 Centre for Translational Microbiome Research, Department of Microbiology, Tumour and Cell Biology, Karolinska Institutet, Stockholm, Sweden,; 6 Department of Head and Skin, Ghent University, Ghent, Belgium,; 7 Kampala City Council Authority Kisenyi Health Center IV HIV clinic, Kampala, Uganda,; 8 Department of Internal Medicine, Case Western Reserve University, Cleveland, Ohio, United States of America

**Keywords:** Type 2 diabetes mellitus, insulin kinetics, field studies

## To the editors of the Pan African Medical Journal

The Makerere University Infectious Diseases Institute (IDI) reported cases of patients presenting with accelerated hyperglycemia following switching to dolutegravir-anchored anti-retroviral therapy (ART) [1]. We sought to characterize glucose metabolism changes in Ugandan people living with HIV (PLHIV) by describing changes in insulin kinetics (insulin secretion, insulin resistance, and insulin clearance) in the first 48 weeks of dolutegravir (DTG)-based ART. One of the methods used to study insulin kinetics is the 75g oral glucose tolerance test (OGTT) with concurrent measurement of insulin and C-peptide in addition to the conventional 0, 30, 60, 90, and 120-minute blood glucose [2,3]. Most insulin kinetic studies have been performed in tailored laboratory and research institutional environments [4,5]. Performance of these studies in very busy field settings can be challenging due to the lack of space, sample processing equipment as well as sample storage facilities. We share our experience performing longitudinal insulin kinetic studies in a high-volume HIV clinic setting in Kampala-Uganda (Kisenyi Health Center IV HIV clinic) with over 12,000 active HIV patients in care.

The glucose metabolism changes in Ugandan HIV patients on dolutegravir (GLUMED) study was a prospective cohort study of 303 HIV-positive anti-retroviral therapy (ART) naive patients recruited between January and October 2021 and followed up for 48 weeks. Serial OGTTs were performed at 0.12 and 36 weeks.

The study nurse counselor called patients in advance to remind them of adhering to an overnight fast of 8-12 hours which was confirmed on arrival at the clinic. Venous access was established with 16-to-18-gauge venous cannulae. Portions of 75g of glucose were pre-packed into disposable cups on site and diluted in 300ml of water on study days, to be taken by the patient within 5 minutes after fasting blood samples were drawn. Serial 5ml blood samples were drawn at 30, 60, 90 and 120 minutes after the 75g glucose load with immediate measurement of blood glucose using ACCU-CHECK™ glucometers from Roche diagnostics. Blood samples were centrifuged in real time and serum samples stored temporarily in duplicate cryovials at -5 to -20°C in the HIV clinic laboratory freezer. At the end of the clinic day, frozen serum samples were transported in cooler boxes with ice packs to be stored at -80°C at the Makerere University Infectious Diseases Institute about fifteen minutes from the study clinic by a laboratory rider. Stored samples were to be used to run C-peptide and insulin assays at the end of the study.

We encountered various challenges in execution of the laboratory processes. There was lack of space to keep patients comfortable during the procedure as well as accommodate the laboratory equipment and personnel. To overcome this, a temporary tent had to be erected to house the study clinician, nurse and laboratory. Additionally, vascular access was hard to establish for some patients. There were occasional blockages of intravenous cannulae with consequent repeated cannulation which was an inconvenience to patients. On a few occasions, patients complained of dizziness and nausea after taking the glucose solution that the test had to be stopped. Despite the challenges, the acceptance rate for the laboratory procedures was high with only one patient dropping out of the study because they had become uncomfortable with the repeated blood draws.

**Funding:** the GLUMED study was funded by: National Institutes of Health (NIH)-Fogarty University of California (UCSF) global health fellowship program, grant number: 2D43TW009343-06; National Institutes of Health, UCSF-Gladstone Center for AIDS Research, grant number: P30AI027763; and the Fogarty International Centre, National Institute of Health (grant# 2D43TW009771-06 “HIV and co-infections in Uganda”).

## Conclusion

Performance of OGTT insulin kinetic studies is feasible even in busy routine ART clinic settings in limited resource settings so long as there is space, trained staff and facilities to ensure proper blood sample handling and storage.
